# Beyond inflammation: the molecular basis of bone remodeling in axial spondyloarthritis and psoriatic arthritis

**DOI:** 10.3389/fimmu.2025.1599995

**Published:** 2025-07-31

**Authors:** Giuseppe Lopalco, Andrea Cito, Florenzo Iannone, Torsten Diekhoff, Denis Poddubnyy, Fabian Proft

**Affiliations:** ^1^ Rheumatology Unit, Department of Precision and Regenerative Medicine and Ionian Area (DiMePRe-J), University of Bari, Bari, Italy; ^2^ Department of Radiology, Charité - Universitätsmedizin Berlin, Humboldt-Universität zu Berlin, Freie Universität Berlin, Berlin, Germany; ^3^ Corporate Member of Freie Universität Berlin and Humboldt-Universität zu Berlin, Department of Gastroenterology, Infectious Diseases and Rheumatology (including Nutrition Medicine), Charite Universitatsmedizin Berlin, Berlin, Germany; ^4^ Epidemiology Unit, German Rheumatism Research Center Berlin, Berlin, Germany

**Keywords:** bone remodeling, axial spondyloarthritis, psoriatic arthritis, cytokines, new bone formation, bone erosion

## Abstract

Spondyloarthritis (SpA) encompasses a group of chronic inflammatory diseases with overlapping genetic, clinical, and radiographic features. Axial spondyloarthritis (axSpA), a subset of SpA, predominantly involves the sacroiliac joints and spine, often progressing to ankylosis, severe disability, and functional impairment. Psoriatic arthritis (PsA), another SpA subtype, is characterized by a heterogeneous phenotype that includes peripheral arthritis, enthesitis, and axial involvement, frequently associated with psoriasis. Bone remodeling in axSpA and PsA is driven by a dynamic interplay between inflammatory cytokines and the uncoupling of anabolic and catabolic processes, resulting in bone erosion, systemic and local bone loss, and pathological new bone formation. In axSpA, tumor necrosis factor-alpha (TNFα) and interleukin-17A (IL-17A) drive osteoclastogenesis via the RANKL pathway while suppressing osteoblast-mediated bone formation through WNT/β-catenin signaling. Mechanical stress, combined with inflammatory mediators, promotes mesenchymal stem cell differentiation and new bone formation, which manifests as syndesmophytes and contributes to progressive ankylosis. Conversely, PsA is distinguished by concurrent bone erosion and neoformation, driven by IL-17A, IL-22, and IL- 23, with axial disease exhibiting asymmetrical, bulky para-syndesmophytes rather than the fine, hair-like syndesmophytes typical of axSpA. Advanced imaging modalities, particularly MRI, have elucidated key mechanisms of disease progression, revealing processes such as fat metaplasia and reparative changes. This review explores the intricate molecular and cellular mechanisms underlying bone remodeling in SpA, emphasizing both shared pathways and disease-specific features. It aims to enhance the understanding of these processes to support the development of more precise and effective therapeutic approaches tailored to axSpA and PsA.

## Highlights

- Bone remodeling in axial spondyloarthritis (axSpA) and psoriatic arthritis (PsA) results from a complex interaction between inflammatory cytokines and mechanical stress, leading to a dynamic balance between bone erosion and pathological new bone formation.
- Key cytokines, including TNF-α and IL-17A, drive osteoclast activation and bone resorption, whereas Wnt/β-catenin signaling modulates new bone formation, with distinct mechanistic differences between axSpA and PsA.
- Advanced imaging techniques, particularly MRI, provide critical insights into disease progression by capturing early inflammatory changes, fat metaplasia, and structural remodeling, thereby improving diagnostic and prognostic accuracy.
- Targeted therapeutic strategies aimed at modulating cytokine pathways (TNF, IL-17, IL-23) hold promise for altering disease trajectories, but precision medicine approaches are essential to address the unique pathophysiological features of axSpA and PsA.

## 1 The landscape of spondyloarthritis

Spondyloarthritis (SpA) represents a heterogeneous group of chronic inflammatory diseases characterized by varying degrees of shared pathophysiological, genetic, clinical and radiographic features (1, 2). A distinguishing hallmark across all subtypes of SpA is the primary involvement of the enthesis, a fibrocartilaginous transitional tissue that anchors tendons and ligaments to bone, conferring resistance to biomechanical stress (3).

Axial spondyloarthritis (axSpA) is a subset of SpA, predominantly (though not exclusively) affecting the spine the spine and sacroiliac joints. Its characteristic progression towards bony ankylosis contributes to profound disability, pain and functional limitations in advanced disease stages (1). According to the 2009 ASAS classification criteria (4), axSpA encompasses both radiographic and non-radiographic forms, distinguished by the presence or absence of structural damage detectable via conventional radiography at the time of assessment. The historical entity of ankylosing spondylitis (AS), previously defined by the New York criteria (5), now aligns with the classification of radiographic axSpA (r-axSpA). The updated classification for non-radiographic disease, enables earlier diagnosis and timely therapeutic intervention. The global prevalence of axSpA is estimated to range between 0.1% to 1.4%, with a male-to-female ratio of approximately 2-3:1 (6).

Within the SpA spectrum, psoriatic arthritis (PsA) constitutes a multifaceted immune-mediated disorder marked by varying incidences of peripheral arthritis, enthesitis, dactylitis and spondylitis, frequently co-occurring with personal or familiar histories of psoriasis. PsA is classified using the CASPAR (7) and typically presents in middle-aged females (8), with an estimated global prevalence of 0.1-1% (9). Axial involvement has garnered increasing attention in recent years. This subset of the disease is more frequently observed in males (8), with prevalence estimates varying widely from 25% to 70% depending on the criteria applied (10, 11). These findings support the recognition of axial PsA as a distinct clinical phenotype. In individuals aged over 60–65 years, this phenotype is associated with more severe clinical and radiographic manifestations and poorer outcomes (12).

Bone involvement in both axSpA and PsA arises from complex interactions between anabolic and catabolic processes, variably contributing to bone loss and new bone formation (13).

Bone loss may manifest as systemic reductions in bone mineral density, thereby increasing fracture risk (14), or as local bone erosions (15), which are more prevalent in peripheral PsA but may also affect the sacroiliac joints and vertebrae in axSpA. Conversely, new bone formation can lead to progressive ankylosis and bridging between adjacent bones, substantially exacerbating disease burden (16).

It is esteemed that up to 66% of patients with axSpA experience intense fatigue, stiffness, limitations in daily activities (including self-care and low-effort tasks), diminished quality of life, higher incidences of nocturnal awakenings, and even severe insomnia (17).

The functional limitations resulting from bone remodeling are directly implicated in occupational impairment (18), with nearly 40% of affected individuals unable to work. Among these, 24% are compelled to take early retirement, while 45% transition to less demanding roles (19).

This review seeks to elucidate the intricate topic of bone metabolism in spondyloarthritis, highlighting shared features and key differences between axSpA and PsA. To this purpose, the latest insights into the radiographic characteristics and underlying molecular pathways of each condition are examined, with the aim of advancing our understanding of the bone-centric pathogenetic mechanisms. Such knowledge may provide clinicians with improved tools to refine therapeutic strategies and enhance disease management.

## 2 Molecular and cellular mechanisms underpinning bone remodeling

The delicate balance of bone tissue homeostasis is maintained through a continuous process of resorption and new bone formation, primarily mediated by osteoclasts and osteoblasts, respectively. The key molecular pathways and therapeutic targets involved in bone remodeling in axSpA and PsA are depicted in **Table 1**. Osteoclastogenesis refers to the process by which osteoblasts (20) directly and indirectly influence the differentiation of macrophage/monocyte precursor cells into active bone- resorbing osteoclasts by producing key signaling molecules (21). The two main cytokines driving this cascade is macrophage colony-stimulating factor (M-CSF) (22) and receptor activator of nuclear factor-kappa B (NF-κB) ligand (RANKL) (23).

**Table 1:** Key molecular pathways and targets in bone remodeling.

Pathway/target	Role in bone remodeling	Therapeutic implications
TNFα	Promotes osteoclastogenesis via RANK-RANKL-OPG axis (55, 56); dual role in MSC activity (61)	TNF inhibitors effective in reducing inflammation and structural progression in axSpA (62) and PsA (98–102)
IL-17A	Drives osteoclastogenesis and MSC differentiation (65); promotes syndesmophytes (66–68)	IL-17 inhibitors halt new bone formation and improve bone density in axSpA (69) and PsA (116–118)
IL-23	Activates entheseal T-cells, promoting IL-17 and IL-22 secretion (112)	Ineffective in axSpA; effective in PsA in radiographic progression (115)
Mechanical Stress (FSS)	Modulates osteocyte activity, reducing RANKL and increasing OPG (79–83)	Potential area for biomechanical therapies to mitigate pathological bone remodeling
DKK1 and Sclerostin	Wnt pathway inhibitors regulating osteoblast activity (35, 36)	Anti-sclerostin therapies (e.g., romosozumab) show promise in enhancing bone formation (37–40)

TNFα, (tumor necrosis factor -alpha); IL-17A, (interleukin 17A); IL-23, (interleukin-23); FSS, (fluid shear stress); DKK1, (Dickkopf-related protein 1); OPG, (osteoprotegerin); RANKL, (Receptor activator of nuclear factor kappa-B ligand); RANK, (Receptor activator of nuclear factor kappa-B); axSpA, (axial spondyloarthtitis); PsA, (psoriatic arthritis).

Osteoclast precursor express RANK on their surface, rendering them responsive to RANKL produced by osteoblast and other cell types, such as osteocytes. This interaction activates multiple intracellular pathways, including NF-kB and MAPK (24).

Recently, significant attention has been given to immunoreceptor tyrosine-based activation motif (ITAM) proteins, which have been shown to play a critical role in inducing calcium signaling within the mineralized matrix. This function is absent in the RANK/RANKL pathway (25). Ultimately, these interactions converge to activate NFATc1, a key transcription factor for osteoclastogenesis. The complexity of this cytokine interplay is further modulated by NFATc1-inhibiting molecules that counteract RANKL activity (26).

RANKL- and RANK-deficient murine models, exhibit marked osteopetrosis due to impaired osteoclast differentiation (27). An important inhibitor of RANK/RANKL pathway is osteoprotegerin (OPG), a soluble decoy receptor for RANKL produced by osteoblasts. OPG suppresses osteoclastogenesis, thereby increasing bone mineral density (28). Engineered mice lacking OPG demonstrate severe osteoporosis (29). Building on these findings, denosumab, anti-RANKL antibody that mimics OPG natural activity, has been developed and is now widely employed in the treatment of osteoporosis and cancer-related bone disorders (30).

Once activated, osteoclasts adhere to the bone surface and initiate the dissolution of hydroxyapatite by creating an acid environment through the secretion of intracytoplasmic hydrogen ions (31). Additionally, the organic components of the bone matrix are remodeled by proteolytic enzymes, such as cathepsin K (27).

Osteoblasts, on the other hand, differentiate from mesenchymal stem cells (MSCs) (32) under the influence of various transcription factors, most notably Wnt proteins and bone morphogenetic proteins (BMPs) (33). Wnt/Wntβ-catenin signaling pathway plays a pivotal regulatory role in new bone formation, which is particularly relevant in inflammatory contexts such as axial spondyloarthritis (34). The equilibrium in bone formation is maintained by two key inhibitors of Wnt pathway: Dickkopf1 (DKK1) (35) and sclerostin (36).

Sclerostin, an antagonist of Wntβ-catenin signaling, is produced by osteocytes (37). Reduced sclerostin levels are associated with increased bone mass (38), making the anti-sclerostin antibody romosozumab, a therapeutic option for diseases characterized by systemic bone loss (39). Recent studies have suggested that cardiotrophin-1 (CT-1), secreted by osteoclasts, suppresses sclerostin production by osteocytes, providing a functional link between bone resorption and new bone formation (40).

Moreover, it is crucial to highlight that sclerostin expression has been shown to be decrease under mechanical stress (41) and in the presence of elevated inflammatory mediators such as interleukin-6 (IL-6) (42) and prostaglandin-E2 (PGE2) (43).

## 3 Imaging features and disease progression in axial spondyloarthritis

**Figure 1:**
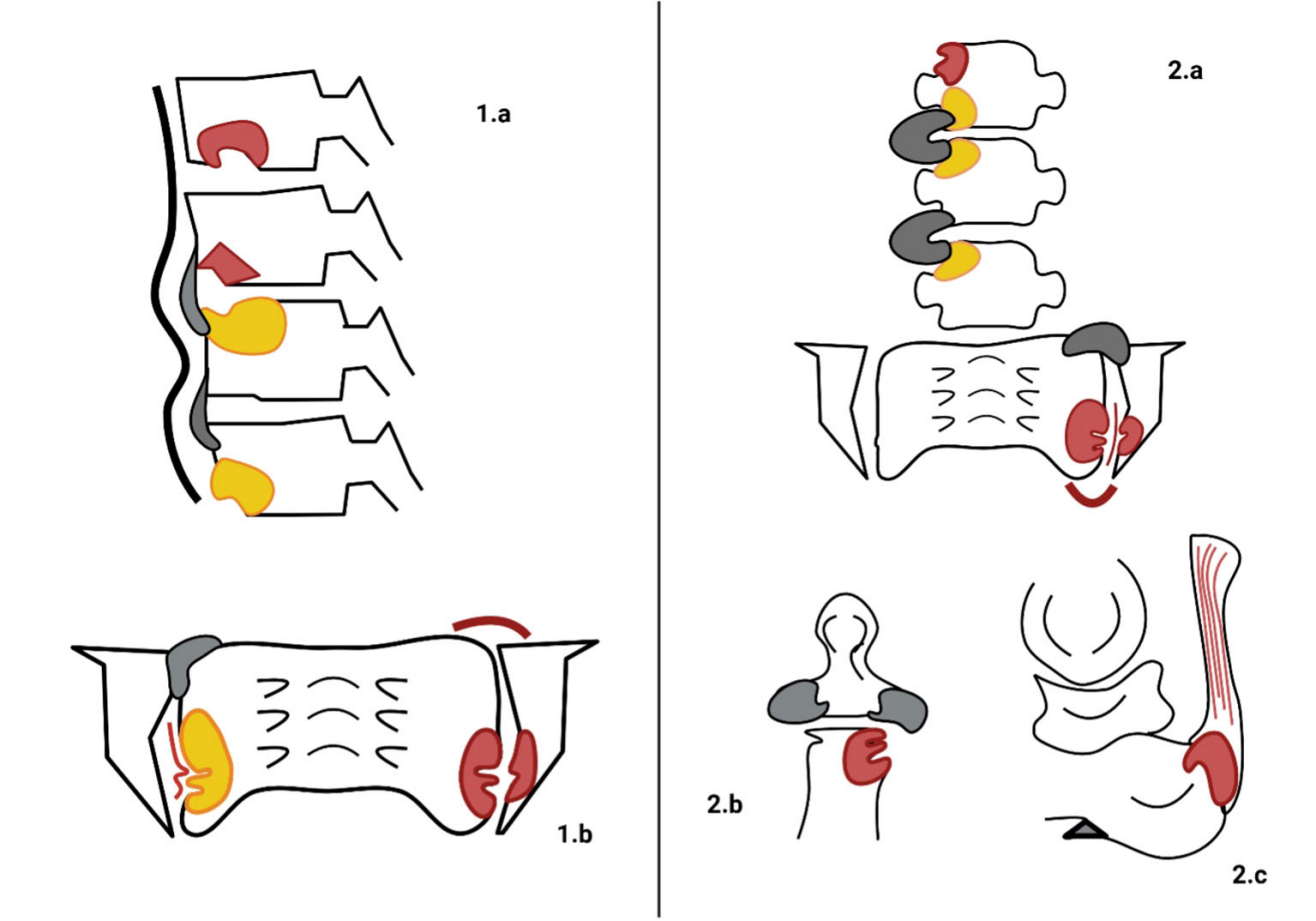
Typical radiographic findings in axial spondyloarthritis (axSpA) and psoriatic arthritis (PsA). On the left, the main radiographic findings of axSpA are illustrated. **(1.a)** depicts vertebral corner erosions with concomitant bone marrow oedema (BMO, red lesions), followed by fat metaplasia (yellow lesions) and progressive new bone formation in the form of thin syndesmophytes (grey sprouts). Additionally, thickening of the anterior ligaments (black wide line) is observed. In **(1.b)**, bilateral involvement of the sacroiliac joints (SIJs) is demonstrated by the presence of BMO, underlying erosions in the lower third, fat metaplasia, backfill of the joint space (thin red line), ankylosis (grey bony sprouts bridging the SIJs), and capsulitis (red curved line). On the right the radiographic characteristics of PsA are stylized. **(2.a)** illustrates wider and thicker bony sprouts (grey sprouts) defining para-syndesmophytes. BMO and fat metaplasia represent two phases of inflammatory involvement of the bone. Monolateral involvement of the SIJ is characterized by BMO, erosions, capsulitis, backfill, and progressive ankylosis. In **(2.b)**, peripheral erosions of the distal interphalangeal joint (DIJ), along with concurrent new bone formation (grey areas), create the characteristic “mouse ears” appearance. In **(2.c)**, Achilles tendon enthesitis (red lesion at the conjunction of the Achilles tendon and calcaneus) and a heel spur (grey triangle-shaped formation sprouting from the calcaneus) are typical findings of peripheral manifestations of PsA.

Axial involvement typically originates in the sacroiliac joints (SIJ), making this the primary focus of diagnostic imaging (44). **Figure 1** (Panel 1.a, 1.b) summarizes the most common radiographic findings observed throughout the disease course. Historically, plain radiography has been the gold standard for assessing bone changes associated with axSpA, with radiographic findings serving as key features in classification criteria for AS. Joint erosion typically occurs in the cartilaginous compartment of the joint while the ligamentous retroarticular space is usually spared. These changes manifest as blurring of subchondral bone on opposing articular surfaces, followed by erosions, joint space widening, and, over time, new bone formation, which appears as articular sclerosis and, eventually, total ankylosis of the joints (45).

However, these radiographic features typically indicate an advanced disease stage, often developing over years after symptoms onset. Consequently, negative radiography will be followed by magnetic resonance imaging (MRI) as the preferred modality for early detection. Although radiography has historically been a cornerstone in diagnosing axSpA, its inability to detect active inflammation in joints and entheses significantly limits its utility in early disease stages. In these instances, MRI offers superior sensitivity and specificity (46).

MRI can reveal active synovitis, periarticular inflammation, tenosynovitis and BMO in axial and peripheral joints as hyperintense signals on T1-weighted images with gadolinium enhancement or short tau inversion recovery (STIR) sequences (46).

On MRI, active inflammation is indicated by bone marrow oedema (BMO), while capsulitis and enthesitis are less specific signs of inflammation. BMO adjacent to the cartilaginous joint surface appears as a high-signal lesion on fat-suppressed T2-weighted (T2w) sequences and a corresponding signal reduction on T1-weighted (T1w) sequences. These findings define osteitis or, when affecting SIJ, sacroiliitis, as outlined in the 2019 update of the ASAS definitions for MRI lesions (47).

The presence of sacroiliitis is a critical criterion for the diagnosis and follow-up of axSpA and is included in the most recent ASAS classification criteria (4).

**Figure 2:**
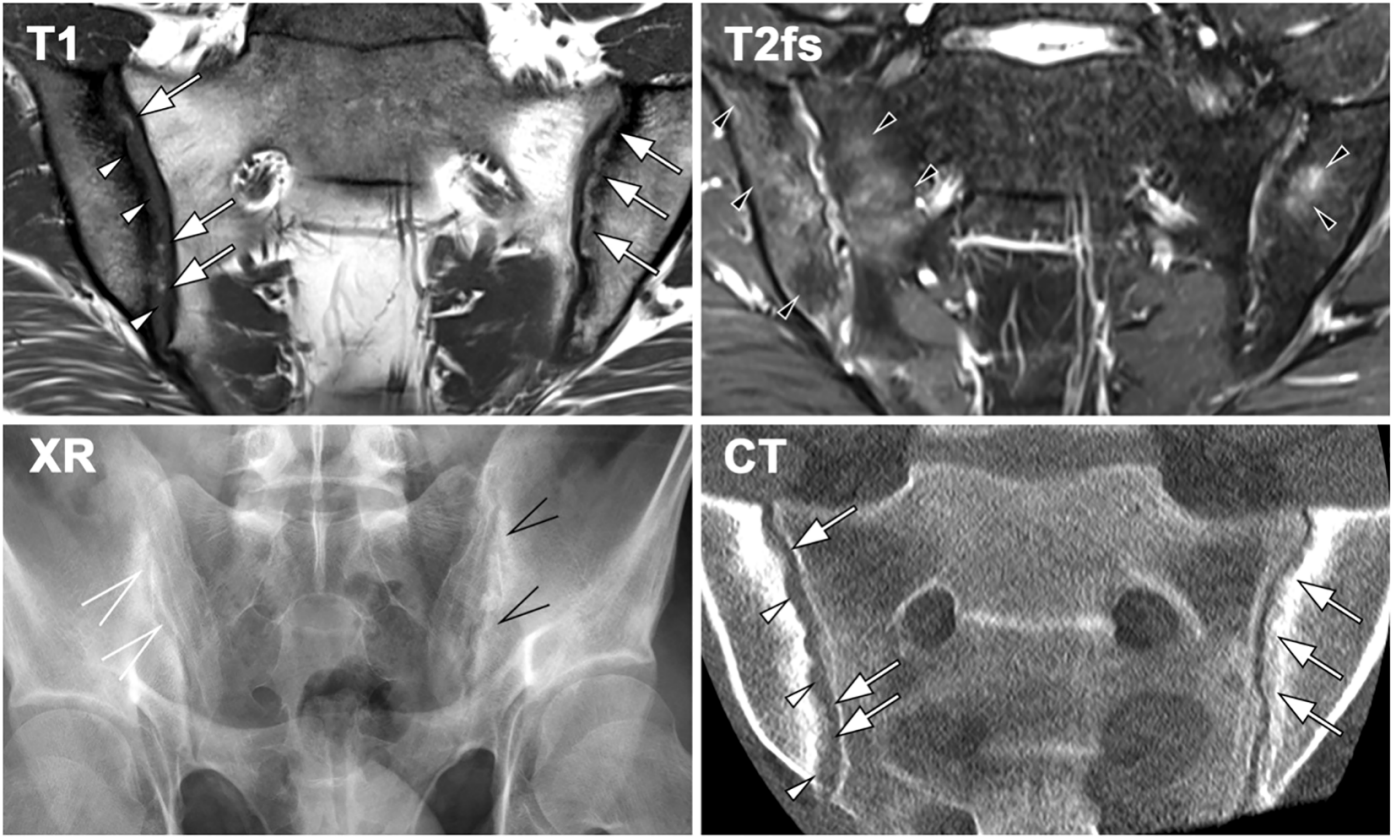
Imaging findings in a 23-year-old patient with sacroiliitis in axial spondyloarthritis (axSpA), demonstrating characteristic features across different modalities. MRI-T1 reveals erosions (white arrowheads) and new bone formation in the form of backfill (white arrows), accompanied by sclerosis. Fat-suppressed T2-weighted MRI detects bilateral bone marrow oedema (BMO, black arrowheads). Radiography (XR) shows erosion and new bone formation on the left sacroiliac joint (SIJ, black open arrows), whereas the right SIJ exhibits only minimal changes, including joint space widening (white open arrows). CT provides comparable visualization of joint erosion (white arrowheads) and new bone formation (white arrows), consistent with MRI-T1 findings.

Bone erosion is another MRI sign of ongoing articular inflammation. It manifests as morphological alterations in the cartilaginous part of the joint, discernible on T1w sequences with and without fat-suppression, and progresses to apparent joint space widening (48). As active inflammation subsides, reparative processes emerge. Fat lesions, visible as bright signals on T1w images, replace areas of BMO. Erosions may become filled with fat metaplasia (backfill), and in advanced stages, bone budding can lead to SIJ narrowing and eventual ankylosis (49) (**Figure 2**).

**Figure 3:**
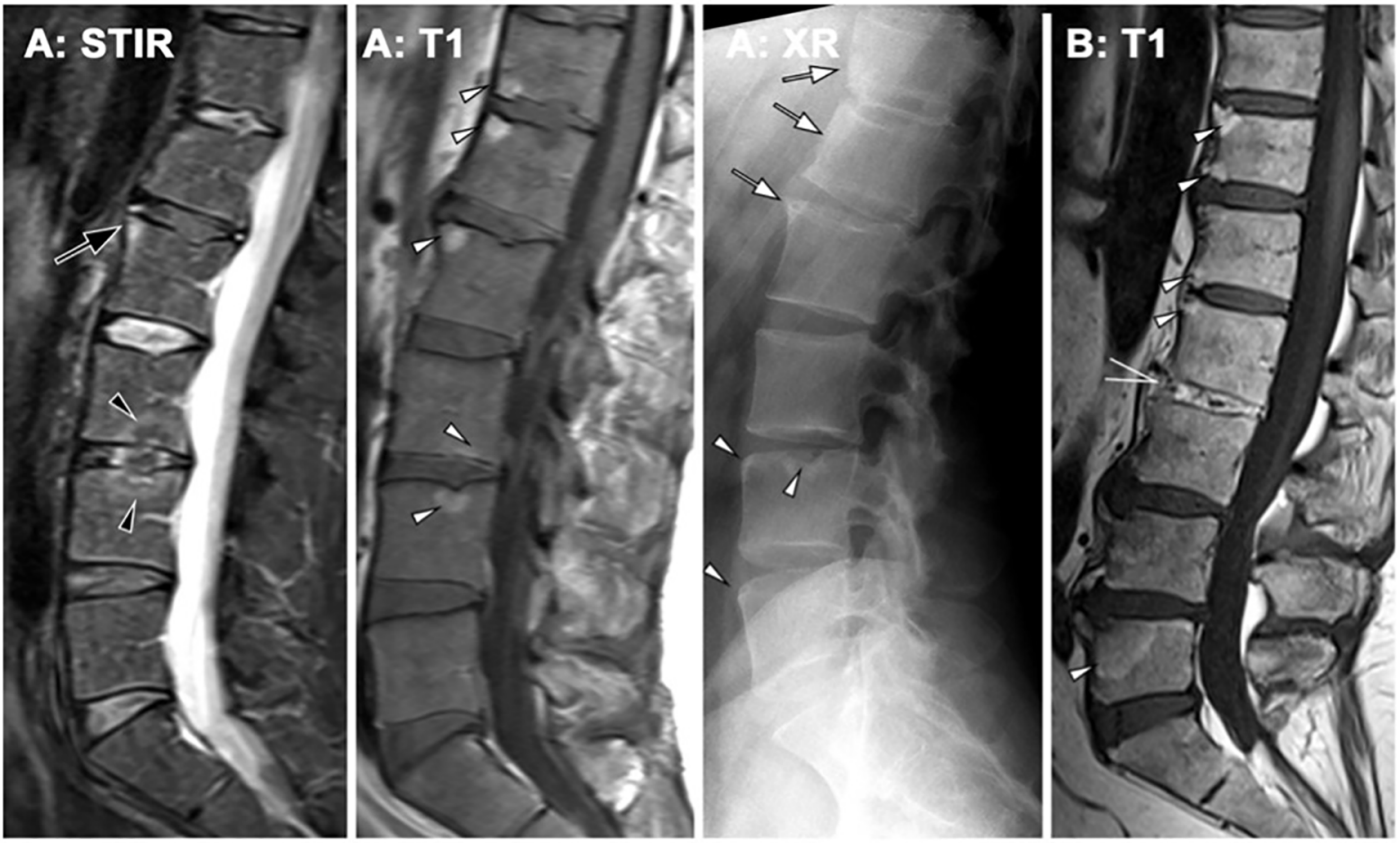
Axial spondyloarthritis (axSpA) at the spine. **(A)** A 42-year-old male patient showing inflammatory corner lesions (black arrow) and endplate lesions (black arrowhead) in STIR and corresponding fat lesions in T1. Radiography (XR) shows new bone formation with box-shaped vertebra and shiny corners (white arrows) and erosions (white arrowheads). **(B)** A different patient with advanced axSpA shows fat lesions (white arrowheads) and transdiscal ankylosis (open white arrow).

A similar pathological sequence occurs in the spine. Active osteitis of the vertebrae is followed by fat metaplasia, corner erosions, and reactive sclerosis, also known as “shiny corner” (45, 50). New bone formation is indicated by the presence of vertebral body squaring or syndesmophytes (51), which are bony outgrowths originating from the attachment sites of the anulus fibrosus at vertebral corners. Syndesmophytes may extend vertically as fine “hair- shaped” projections along the anterior vertebral margin. They may remain non-bridging (52) or, in chronic cases, fuse adjacent vertebrae (bridging-syndesmophytes), often accompanied by similar fusion processes in the facet joints, culminating in the classic “bamboo spine” appareance (51, 53). Inflammation of the vertebral endplates (so-called Andersson lesions) may additionally result in transdiscal ankylosis, best depicted by fat signal inside the disc space on MRI (**Figure 3**).

### 3.1 The pathophysiology of bone remodeling in axial spondyloarthritis

In axSpA, chronic inflammation primarily drives bone resorption, while new bone formation is believed to arise from reparative processes occurring in the context of chronic relapsing-remitting disease. MRI studies have demonstrated that the degree of spinal inflammation correlates negatively with bone mineral density (54), underscoring the interplay between inflammation and bone pathology in axSpA.

Among inflammatory mediators, tumor necrosis factor-α(TNFα) plays a pivotal role in the bone remodeling processes. TNFα directly and indirectly activates osteoclasts and promotes osteoclastogenesis via the RANK-RANKL-OPG axis.

Specifically, TNFα induces RANKL expression in osteoblasts, T-cells and B-cells (55), while concurrently reducing OPG levels (56). Moreover, TNFα enhances RANK surface expression on osteoclast precursors, amplifying bone resorption (55).

Animal studies on murine models suggest that TNFα may also influence new bone formation by inducing DKK1 (57), a potent inhibitor of Wnt/β-catenin pathway. This dual role, promoting bone resorption while inhibiting neoformation, gave rise to the “TNF-brake” hypothesis (58). However, serum levels of DKK1 in axSpA patients are paradoxically lower than in healthy individuals, as are sclerostin levels, another Wnt pathway inhibitor (59). This suggests that TNFα may have a relatively limited role in activating the DKK1-sclerostin axis in axSpA (60).

TNFα exhibits context-dependent effect on MSCs. In the presence of abundant osteoclasts, TNFα inhibits osteoblastogenesis. Conversely, in their absence, TNFα can stimulate MSC differentiation. This dual effect may partially explain the enhanced new bone formation observed in axSpA, especially at spinal entheses and SIJs, where resident osteoblasts appear highly responsive to TNFα activation (61). Imaging studies using positron emission tomography/MRI have demonstrated reduced osteoblasts metabolic activity in the SIJs and spine of patients treated with TNFi (62), further highlighting TNFα role in new bone formation. Accordingly, TNF inhibitors have been shown to reduce spinal radiographic progression in first in axSpA within the first two years of therapy (63).

The apparent dichotomy between bone erosion and new bone formation may be explained by differential activity of soluble TNF and transmembrane TNF (tmTNF). Soluble TNF is likely responsible for systemic bone loss, whereas tmTNF, predominantly expressed in synovial tissues, may drive ossification process (64).

Inteleukyn-17A/F (IL-17A/F) has emerged as another key mediator of bone remodeling in axSpA. IL-17A enhances RANK expression on osteoclast precursors, thereby promoting osteoclast differentiation and bone resorption (65). Inhibition of IL-17A with secukinumab has been associated with increased BMD in the lumbar spine of axSpA patients (66).

Interestingly, IL-17A may also contribute to syndesmophyte formation, suggesting differential effects in trabecular bone versus entheses. IL-17 A stimulates osteoblast differentiation, mineralization of osteoid matrix, and bone metabolism, as reflected by increased levels of alkaline phosphatase (ALP) (67) and lower concentrations of Wnt inhibitors such as sclerostin and DKK1 (68). Prolonged secukinumab therapy has been shown to be able of achieving similar positive results on slowing radiographic progression as adalimumab at week 104 (69).

Dual inhibition of IL-17A/F, as achieved with bimekizumab, has demonstrated even greater suppression of new bone formation compared to IL-17A inhibition alone (70).

This effect may be mediated by IL-17A produced by γδ T -cells (γδ-Tc), which are highly concentrated in spinal entheseal and peri-entheseal tissues and appear to drive MSCs differentiation and new bone formation (71). Importantly, γδTc cytokine production is largely independent of IL-23 (72).

JAK2 inhibition has also been shown to reduce ALP levels in AS patients, suggesting that IL- 17-dependent osteoblast activation relies heavily on the JAK/STAT3 pathway (67). In murine models JAK inhibition suppressed inflammation and prevented both periosteal and entheseal new bone formation (73), further implicating this pathway in axSpA pathophysiology.

The interplay between IL-6, parathyroid hormone-related protein (PTHrP), prostaglandin E2 (PGE2), and JAK/STAT signaling also contributes to bone remodeling. IL-6 is directly implicated in bone resorption through its interaction with receptors expressed on the membranes of pre-osteoclasts and osteoblasts. Upon binding, IL-6 triggers osteoclast differentiation (74) and activated the JAK-STAT3 signaling pathway in osteoblasts, ultimately promoting the production of pro-osteoclastogenic mediators, including RANKL, PTHrP and PGE2 (75). Within the bone marrow microenvironment, PTHrP and PGE2 further enhance RANKL and IL-6 release from osteoblasts, demonstrating their autocrine regulatory capabilities (75).

Despite the initial promise of targeting IL-6 in bone resorption, clinical therapies aimed at IL- 6 inhibition have shown limited effects on bone mass (76). Nevertheless, IL-6 blockade has demonstrated positive effects on bone mass (77), likely due to its impact on bone resorption and its role as a mediator of inflammation. Supporting this, decreased serum levels of DKK1 have been observed in patients undergoing anti-IL-6 treatment (78).

Mechanical stress is now widely recognized as a critical regulator of bone remodeling. Osteocytes, acting as mechano-sensors within mineralized lamellae, detect mechanical stimuli through their cilia and dendrites, subsequently modulating the composition of extracellular matrix (79). Osteocyte activity results in the formation of lacunae and canaliculi, empty and fluid- filled spaces within the mineralized matrix, that generate fluid shear stress (FSS), establishing a mechanobiological link between mechanical stress and bone tissue activity (80).

*In vitro* studies have demonstrated that increased FSS is associated with reduced RANKL gene expression and increased OPG levels, contributing to enhanced BMD. Conversely, reduced mechanical stress leads to osteocyte necrosis, releasing damage-associated molecular patterns (DAMPs) that activate osteoclasts and underscore the significance of the osteocyte-osteoclast axis in bone homeostasis (81). Furthermore, FSS has been shown to directly modulate the differentiation of both osteoclasts (82) and osteoblasts (83) from their precursor cells.

**Figure 4:**
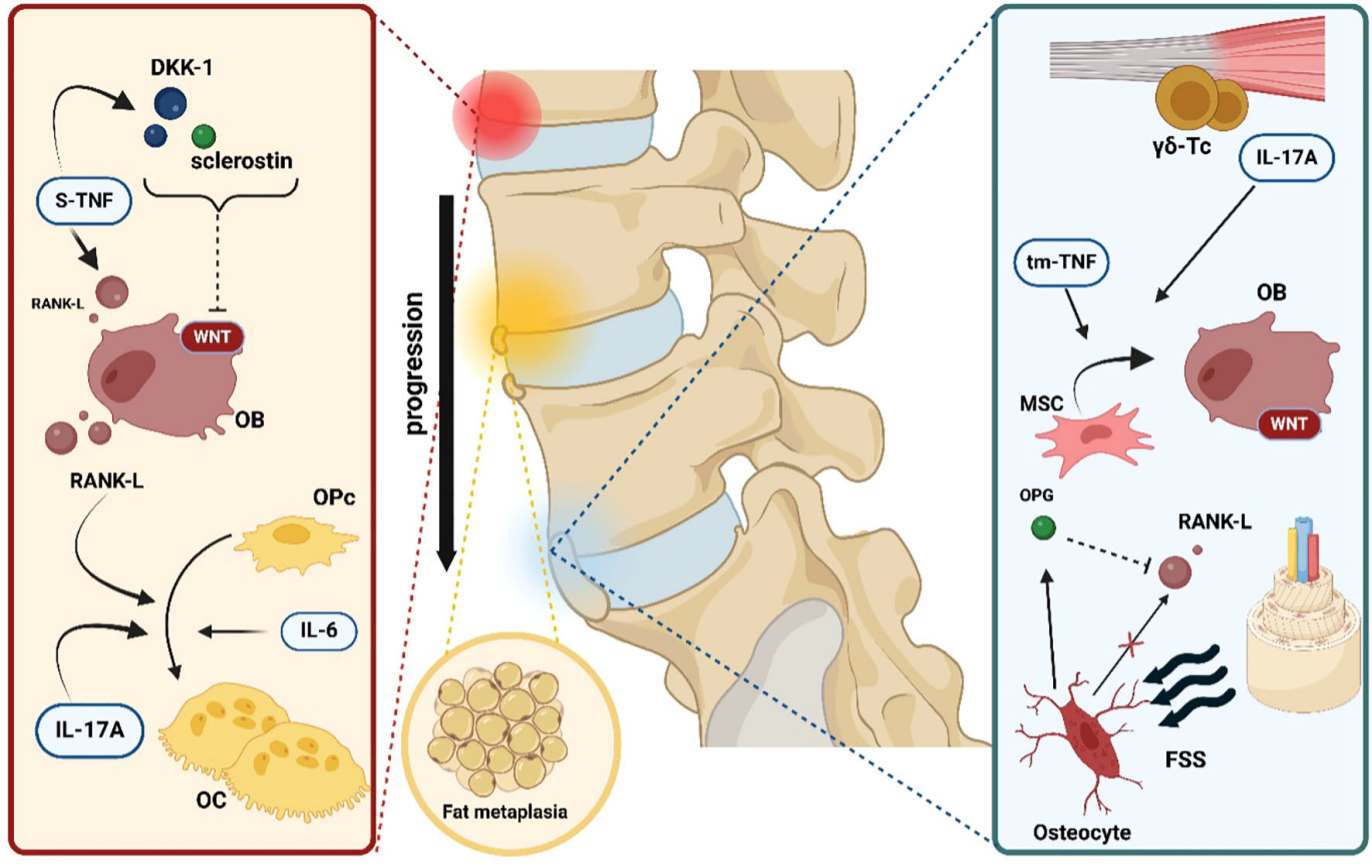
Key mechanisms of bone pathology and remodeling in axSpA. The first step in bone pathology in axial spondyloarthritis is represented by local inflammation. Soluble tumor necrosis factor (s-TNF) enhances osteoclast (OC) differentiation from osteoprogenitor cells (OPc) by inducing receptor activator of nuclear factor kappa-B ligand (RANK-L) expression on osteoblasts (OB). In addition, s-TNF increases the concentrations of both dickkopf-related protein 1 (DKK-1) and sclerostin, two powerful inhibitors of the WNT/β- catenin pathway, thus slowing new bone formation. Interleukin-17A (IL-17A) and interleukin- 6 (IL-6) exert a direct positive effect on osteoclastogenesis. The next pathophysiological step is represented by fat metaplasia filling the gaps created by the ongoing erosive inflammatory process. Finally, in the microenvironment of the sacroiliac and spinal joint’s enthesis, resident gamma-delta T cells (γδ-Tc) independently produce IL-17A, which is capable of driving mesenchymal stem cell (MSC) differentiation into active OB. Moreover, another form of tumor necrosis factor, transmembrane tumor necrosis factor (tm-TNF), has been shown to positively influence osteoblastogenesis. Lastly, increased fluid shear stress (FSS) is detected by osteocytes, which act as mechanoreceptors and respond by stimulating new bone formation via the reduction of RANK-L gene expression and, conversely, the augmentation of osteoprotegerin (OPG) levels.

A theoretical model of bone remodeling in axSpA emerges from evidence linking disease activity with BMD alterations. **Figure 4** shows the key mechanisms of bone remodeling in axSpA, illustrating the interplay between inflammation, bone resorption, and pathological new bone formation. Increased Bath Ankylosing Spondylitis Metrology Index (BASMI) (84) scores correlate significantly with lower systemic BMD (85). Similarly, syndesmophyte height is a strong predictor of reduced BMD, trabecular strength, and overall bone integrity (86). AxSpA patients with syndesmophytes and articular bone bridging face an increased risk of fractures associated with osteoporosis (87).

It has been hypothesized that systemic and local reductions in BMD due to inflammation may directly contribute to syndesmophyte formation by increasing the mechanical burden on weakened vertebrae (88). Newly formed bone bridging then reduces mechanical loading on trabecular bone, perpetuating additional bone loss. Additionally, fat metaplasia at previously inflamed lesion sites appears to promote new bone formation, further exacerbating the pathological cycle of bone remodeling in axSpA (89).

## 4 Imaging features and disease progression in psoriatic arthritis

PsA is characterized by the involvement of bone tissues in both axial and peripheral joints. A key radiographic hallmark of the disease is the pronounced new bone formation, often resulting in a “fuzzy” appearance of the juxta-articular bone (90), which is incorporated into the CASPAR classification criteria (7).

**Figure 5:**
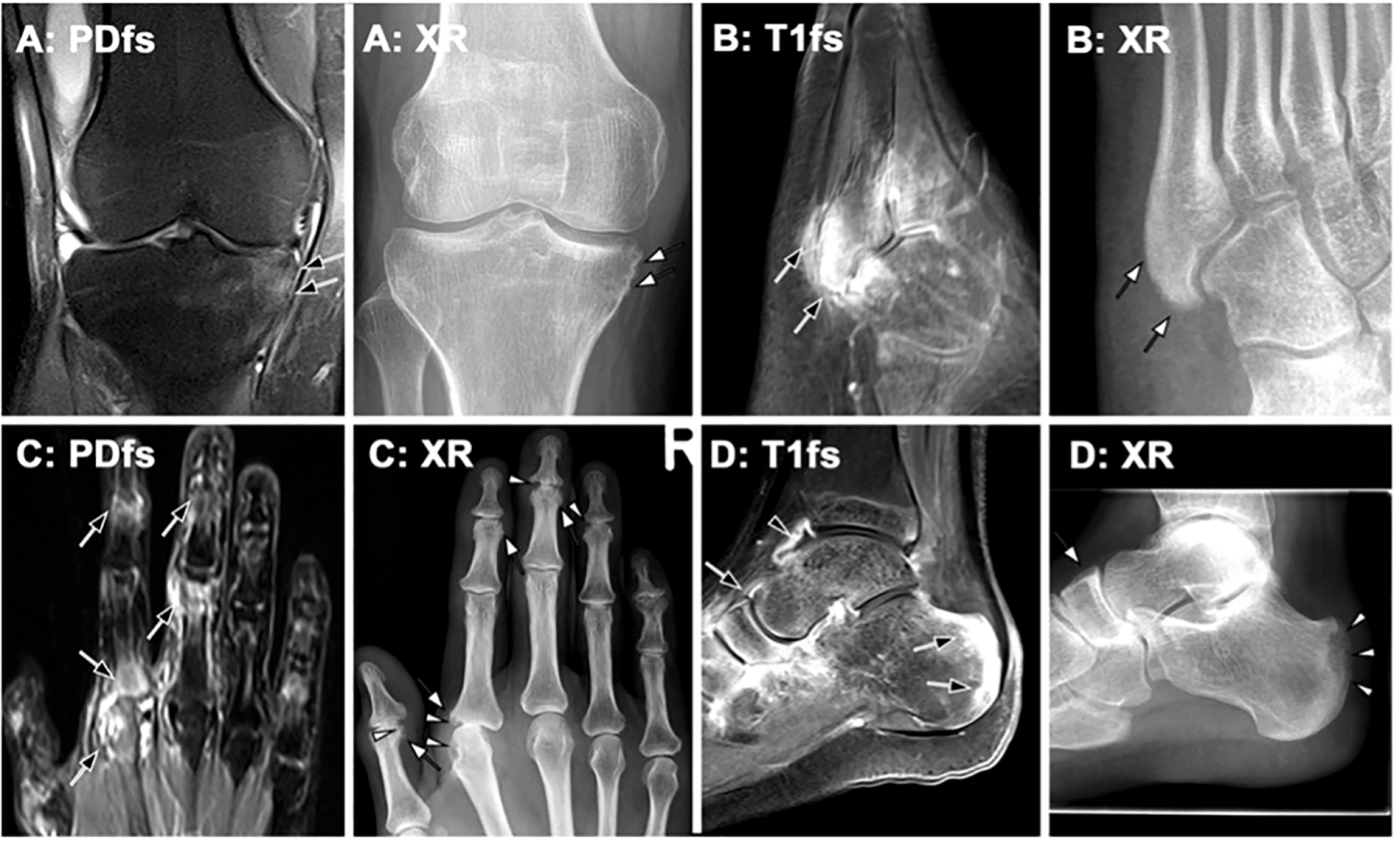
Imaging findings in peripheral psoriatic arthritis (PsA)– correlation of MRI and radiography (XR). Representative imaging findings in different patients with PsA, demonstrating MRI features and their progression on follow-up radiography. **(A)** Knee MRI of a 35-year-old male patient with PsA shows enthesitis at the medial tibia. Radiography obtained two years later reveals new bone formation in the same area (white arrows). **(B)** An 18-year-old male patient with PsA exhibits marked enthesitis on post-contrast MRI (black arrows). Four years later, follow-up radiography demonstrates pronounced periosteal ossification (white arrows). **(C)** In a 54-year-old patient with PsA, MRI shows inflammatory changes at multiple joints (black arrows). Radiography performed only a few months later depicts both erosions (white arrowheads) and new bone formation (white arrows). **(D)** MRI reveals synovitis (black arrowhead) and enthesitis (black arrows). One year later, follow-up radiography shows significant erosion (white arrowheads) and periosteal proliferation (white arrow).

**Figure 6:**
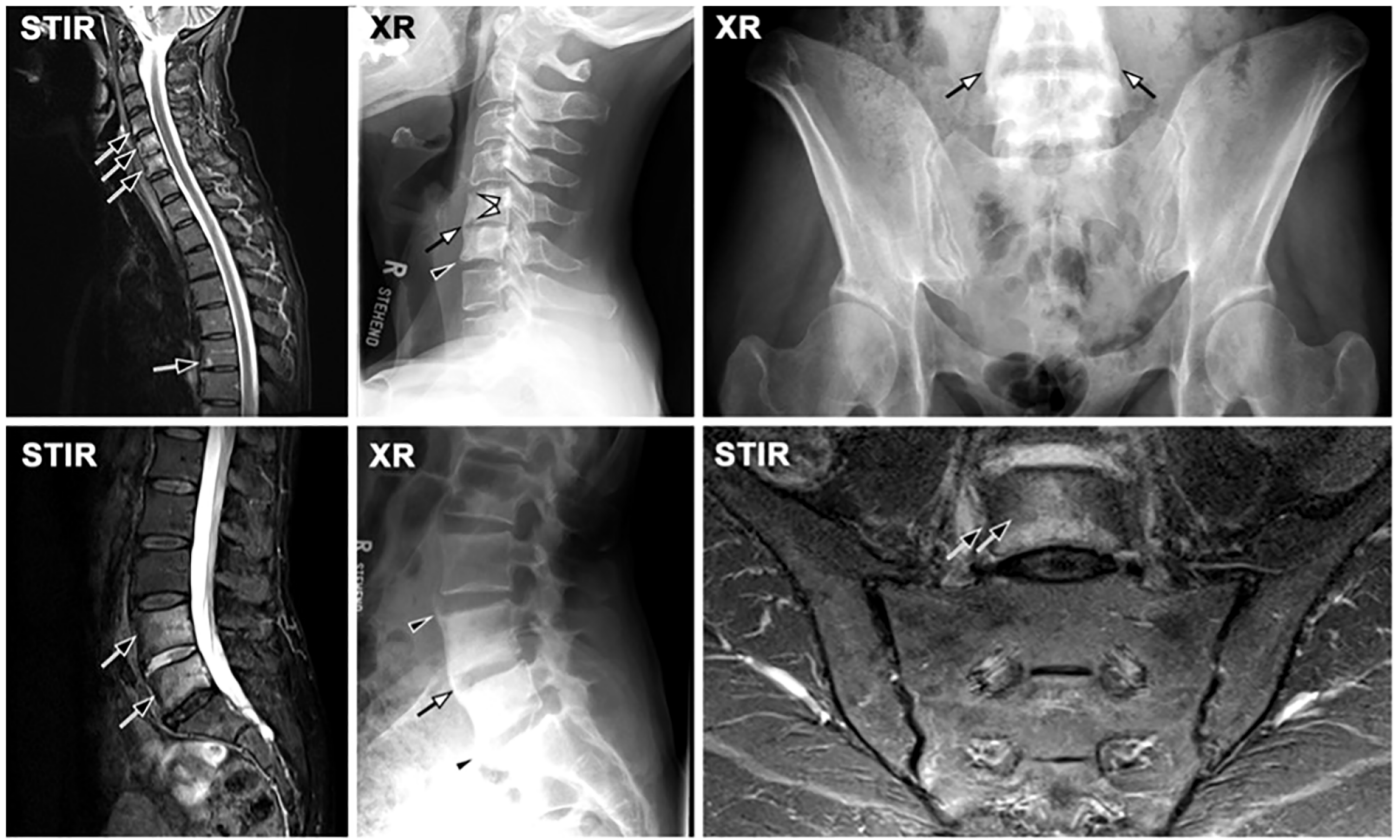
Axial psoriatic arthritis (axPsA) without involvement of the sacroiliac joints (SIJ). MRI-STIR shows active inflammation in several levels of the spine (black arrows), corresponding to erosion (white arrowheads), bridging (white arrows) and non-bridging (black arrowheads) bulky syndesmophytes. Notably, the SIJ is fairly unremarkable.

In peripheral joints, notable radiographic features include metaphyseal periostitis, which manifests as periosteal new bone layering or irregular cortical thickening. In some cases, this may culminate in the so-called “ivory phalanx” sign, where an entire phalanx displays increased radiodensity. Conversely, ongoing marginal erosions and bone resorption may lead to substantial alterations in joint integrity, including subluxations and the characteristic “pencil- in-cup” deformity (91). These two distinct aspects of bone pathology, new bone formation and bone resorption, often coexist, contributing to the overall burden of the disease and negatively affecting the patient’ s clinical progression and treatment outcomes (**Figure 5**). In axial PsA, sacroiliitis is a hallmark feature, sharing similarities with axSpA. It is defined by the progressive development of erosions, sclerosis, bony bridges, and ankylosis, as per the New York criteria (5). The rest of the axial skeleton may be the initial site of disease manifestation, independent of prior SIJ inflammation (**Figure 6**). Axial involvement is typified by neo-appositional changes such as ligamentous ossifications, ossification of anterior vertebral ligaments, squaring of vertebral bodies, and irregular, bulky syndesmophytes (para-syndesmophytes). Simultaneously, erosive lesions of vertebral surfaces are commonly observed (92). **Figure 1** (panel 2.A) illustrates para-syndesmophytes, along with the two phases of bone inflammation, and unilateral sacroiliac joint involvement with progressive changes. Panel 2.B shows distal interphalangeal joint alterations, while panel 2.C depicts Achilles tendon involvement, both characteristic of PsA.

### 4.1 The pathophysiology of bone remodeling in psoriatic arthritis

**Figure 7:**
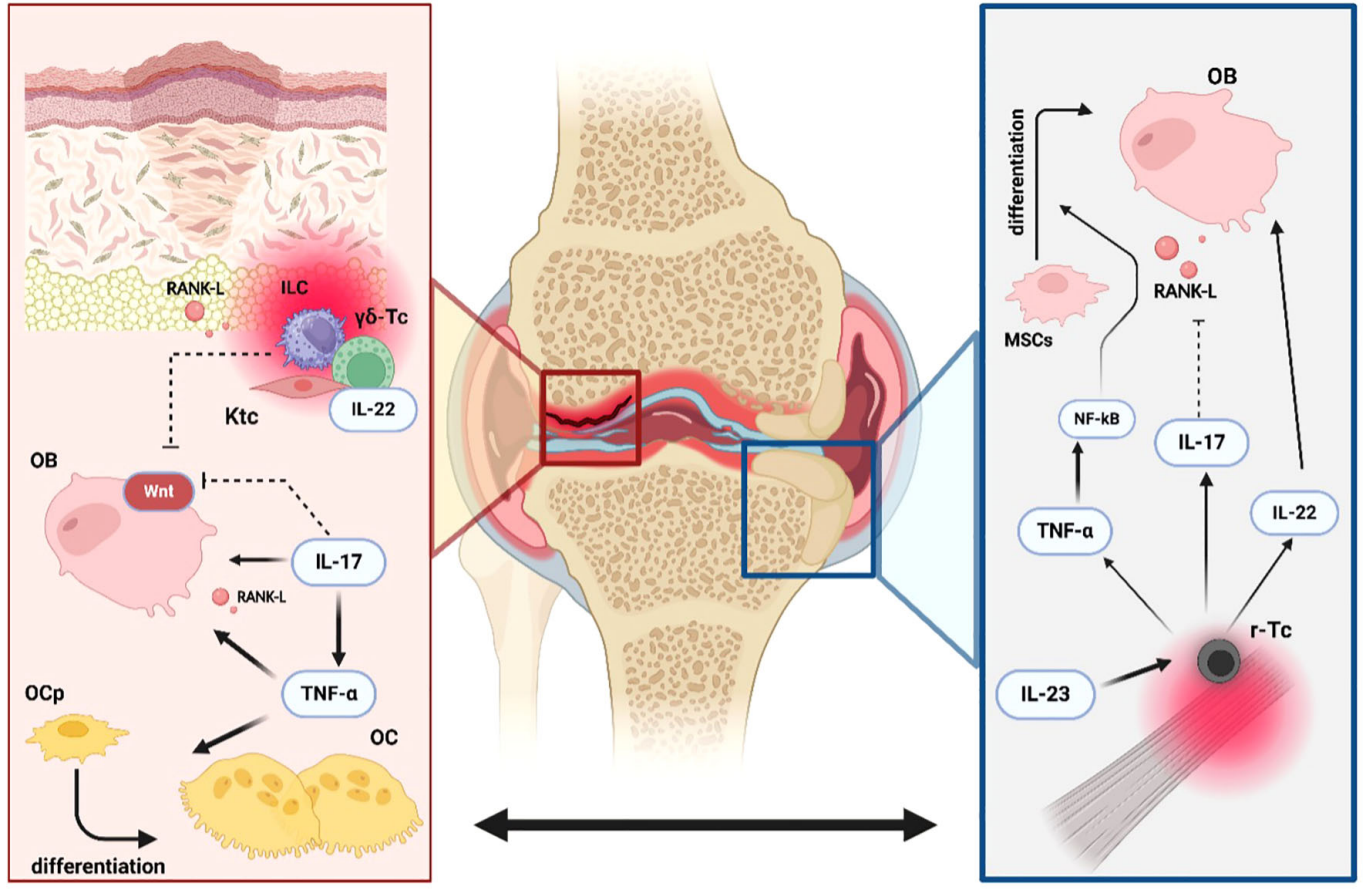
Dual processes of erosion and new bone formation in PsA. Erosions and new bone formation are processes that occur simultaneously in Psoriatic Arthritis (PsA). Tumor necrosis factor-alpha (TNFα) enhances the differentiation of osteoprogenitors (OPc) into mature osteoclasts (OC) both directly and indirectly by increasing the production of receptor activator of nuclear factor kappa-B ligand (RANK-L) by osteoblasts (OB). Interleukin-17A (IL-17A) promotes osteoclastogenesis by inducing RANK-L expression on OB while exerting an inhibitory effect on the WNT signaling pathway. Resident innate lymphoid cells (ILC3s), gamma-delta T cells (γδ-Tc), and keratinocytes (Ktc) in psoriatic plaques have demonstrated the ability to create a pro-erosive environment in peripheral joints by increasing local production of RANK-L, IL-22, and by blocking the WNT signaling pathway. Simultaneously, entheseal resident T-cells (r-Tc), under the influence of interleukin- 23 (IL-23), drive the characteristic new bone formation observed in PsA. Elevated levels of TNFα, IL-17A, and IL-22 promote osteoblast activity by activating the nuclear factor-kappa B (NF-κB) pathway in mesenchymal stem cells (MSCs), inhibiting RANK-L production, and directly stimulating osteoblastogenesis, respectively.

Bone metabolism in PsA, as in other arthritis models, is profoundly influenced by inflammatory cytokines that modulate the activity of various cellular populations involved in bone homeostasis. **Figure 7** shows the key mechanisms of bone remodeling in psoriatic arthritis (PsA), illustrating the interaction between inflammation, osteoclast activation, and new bone formation driven by immune cells and cytokine signaling.

One of the key mediators in PsA pathogenesis, TNF, plays a pivotal role in osteoclastogenesis by inducing RANKL expression (93). *In vitro* studies suggest that TNFα may also exert direct effects independent of RANKL. Prolonged exposure to TNFα has been shown to enhance osteoclast progenitor differentiation (94), while TNF-deficient mouse models exhibit reduced levels of transcription factors such as NFAT1c in myeloid cells (95), leading to impaired osteoclast recruitment, differentiation, and bone resorption. The role of TNFα in PsA is further supported by phase III clinical trials of anti-TNF agents, which demonstrate reduced radiographic progression and decreased osteoclast progenitors’ levels in peripheral blood (96). All five approved anti-TNF agents for PsA have consistently shown efficacy in halting or slowing radiographic progression over two years (97–101).

While TNFα is typically associated with inhibition of osteoblast differentiation through suppression of insulin-like growth factor (IGF-1) (102), low doses of TNFα have been found to activate NF-kB signaling in mesenchymal stem cell (103), promoting osteoblast differentiation and favoring bone formation. Consistently, TNFα administration in fractured mice accelerated healing (104).

Similar dual roles have been observed with IL-17A. *In vitro*, IL-17A promotes osteoclastogenesis (105) by RANKL expression on osteoblasts and stimulating osteoblast secretion of resorptive cytokines such as TNFα, PGE2 and IL-1 (106).

Additionally, IL-17A inhibits WNT signaling in osteoblasts (107), suppressing bone formation. However, high concentrations of IL-17A have been shown to reduce RANKL expression and decrease bone remodeling markers such as MMPs and cathepsin K (108). Furthermore, IL-17A induces a pro-oxidative environment in bone marrow, enhancing MSCs differentiation into mature osteoblasts (109).

The interplay between IL-17A and TNFα further modulates bone metabolism. IL-17A enhances TNFα-dependent matrix deposition by MSCs while mitigating TNFα-induced BMP feedback- inhibition, promoting new bone formation in the absence of osteoclasts. Conversely, IL-17A and TNFα synergistically upregulate Schnunri-3 finger protein (110), a key regulator of ossification, which drives osteoclast activity and local bone resorption (111).

*In vivo* studies have demonstrated that the expression of IL-23 in murine models induces enthesitis by activating IL-23 receptor expressing entheseal resident T-cells. Activation of this cell lineage leads to increased levels of TNFα, IL-17, and IL-22, which drive entheseal and periosteal new bone formation while concurrently exhibiting erosive features (112). Notably, IL- 22 alone has been identified as a potent up-regulator of osteoblast activity, whereas its co- presence with TNF suppresses this effect (113).

A well-established pathogenetic pathway in PsA is the connection between skin inflammation and joint disease. In this context, IL-22 dual role in bone metabolism has garnered increasing attention due to its pivotal role in psoriatic skin inflammation (114). Skin resident γδ-Tc, innate lymphoid cells (ILCs) and keratinocytes, activated within the inflammatory milieu of psoriatic plaque, have shown enhanced local production of RANKL and anti-Wnt activity, promoting osteoclastogenesis and, consequently, bone resorption. In murine PsA models, increased epithelial expression of IL-17A has been shown to progressively induce systemic and local bone loss (107).

Given these findings, the development of therapeutic agents targeting IL-17 and anti-IL23 has been met with significant anticipation. Combined analyses of the PSUMMIT 1 and 2 trials yielded contrasting results, with the anti-IL12/23 monoclonal antibody ustekinumab proved to inhibit radiographic progression in TNF naïve patients, but not in those previously treated with TNFi (115). Additional data are required to evaluate the impact of the anti-IL23 agent guselkumab on bone metabolism.

More promising outcomes have been observed with anti-IL17A monoclonal antibodies, such as secukinumab (116) and ixekizumab (117), both of which demonstrated reduced rates of radiographic progression in PsA cohorts. Additionally, the recently approved IL-17A/F inhibitor bimekizumab, has shown encouraging results. In phase III randomized controlled trials, bimekizumab improved SIJ inflammation, as assessed by MRI (118).

PsA synovial tissues analyses reveal higher levels of IL-6 compared to healthy controls (119). IL-6 promotes bone remodeling by enhancing osteoclast differentiation through a non- RANKL-dependent mechanism (120). Further investigations in murine models corroborated these findings, suggesting that IL-6 acts synergistically with TNFα as a potent inducer of osteoclastogenesis, even in the absence of osteoblasts (121). However, the anti-IL-6 receptor monoclonal antibody tocilizumab failed to demonstrate significant clinical benefits in PsA patients (122). Other mediators of bone metabolism have also been implicated in PsA. Bone morphogenetic proteins and DKK-1 show impaired concentrations in PsA patients. Elevated BMP levels, particularly BMP-7, were observed in ankylosed SIJ enthesis in murine PsA models (123) and correlated with lower limb enthesitis (124). Conversely, DKK-1 serum concentration data are inconsistent; some studies report significantly lower levels in PsA patients compared to those with rheumatoid arthritis (125), while others suggest increased DKK- 1 levels in PsA cohort (126).

## 5 Shared pathways and distinct mechanisms in bone remodeling of axSpA and PsA

AxSpA and PsA share a fundamental characteristic: both involve local inflammation of joints and periarticular tissue. The cytokines driving this inflammation exert extensive effects on bone remodeling. Both conditions are characterized by systemic and local bone loss alongside a pro-ankylosing tendency, though the patterns of these changes differ significantly between the two diseases (45–53, 90–92).

Key mediators such as TNFα, IL-17, and IL-6 play critical roles in driving the distinctive bone lesions that are hallmarks of axSpA and PsA. While the relative contributions of these cytokines to the pathological processes are not yet fully elucidated, it is evident that their interplay underpins the complex regulation of tissue changes, including erosions and new bone formation (56–72, 93–118).

Despite sharing a common cytokine milieu involved in bone remodeling, axSpA and PsA exhibit profound differences in their morphological, pathogenetic, and topographic characteristics. AxSpA is marked by axial bony neo-apposition, which ca culminate in complete joint ankylosis. In the early phases erosive lesions predominantly affect axial skeleton, representing scars of intense inflammation targeting the joints. These erosions lead to subtle alterations in bone structure, which in turn disrupt mechanical force distribution and activate a feedback loop involving osteoblasts, osteocytes and osteoclasts. New bone formation in axSpA appears to be a reparative response to inflammatory or mechanical injury, aiming to restore an impaired weight-bearing system (88, 89).

Mechanical stress may also act as a potent activator of γδ-Tc within spinal entheseal and peri- entheseal tissues, driving IL-23-independent IL-17A production, osteoblast activation, and subsequent new bone formation in forms such as fine hair-shaped syndesmophytes, bone buds, and eventual ankylosis (66–73). Furthermore, syndesmophyte formation contributes to trabecular bone unloading, which suppresses osteocyte activity and exacerbates bone loss, perpetuating a vicious cycle (50–84).

In contrast, PsA primarily shows a peripheral erosive pattern, likely influenced by the close interplay between joint and skin inflammation. Psoriatic plaques, through skin resident γδ-Tc, ILCs and keratinocytes activated in the inflammatory milieu, promote enhanced osteoclastogenesis and local and systemic bone loss (107, 113).

A key distinction in PsA lies in the uncoupling of osteoblasts and osteoclasts activity. Unlike axSpA, erosive lesions and new bone formation in PsA are not chronologically linked and may occur simultaneously. Moreover, axial bone neo-apposition in PsA differs significantly from that in axSpA, producing bulkier, asymmetrical para-syndesmophytes, indicative of a more chaotic new bone formation process. This distinction likely reflects differences in the cytokine’s networks driving disease processes (91–121).

AxSpA appears to be predominantly influenced by TNFα, as suggested by the “TNFα brake hypothesis”, which posits a more controlled balance between osteoblast and osteoclast activity, leading to a more structured bone formation process (58). In contrast, axial bone remodeling in PsA shows reduced sensitivity to TNFα effects and greater reliance on IL-22, IL-23, and IL-17 activity, resulting in a less regulated remodeling process. Myeloid cells resident in peripheral entheses of axPsA patients exhibit increased IL-23 production (127), further highlighting this molecular disparity.

A comparative overview of bone remodeling mechanisms, structural features, and therapeutic effects in axSpA and PsA is provided in **Table 2**. This divergence in cytokine activity is clinically underscored by the differential efficacy of anti-IL23 agents. Phase III trials of IL-23 inhibitors such as ustekinumab (128), risankizumab (129), and guselkumab (130) failed to demonstrate efficacy in axSpA patients. Conversely, these agents demonstrated significant benefits in PsA cohorts, including positive effects on axial radiographic progression (131–133).

**Table 2:** Bone remodeling pathways, structural damage, and therapeutic modulation in axSpA and PsA.

	axSpA	PsA
MAIN PATHOGENIC BONE REMODELING PATHWAYS	◼ In spinal entheses, osteoblasts and γδ T cells are highly responsive to TNF-α (61) and produce IL-17 independently of IL-23 signaling (71) ◼ Mechanical stress further contributes to pathological new bone formation (83, 88, 89)	◼ Resident myeloid cells at peripheral entheses exhibit increased IL-23 production (127) ◼ IL-23 receptor–expressing entheseal T cells enhance the production of IL-17, IL-22, and TNF-α (112) ◼ Skin-resident γδ T cells, ILC3s, and keratinocytes from psoriatic plaques enhance the local production of RANKL and inhibitors of the Wnt signaling pathway (107)
CHARACTERISTICS OF BONE REMODELING	◼ Erosions temporally precede and contribute to new bone formation by altering spinal biomechanical homeostasis (88, 89) ◼ Reparative phase leading to new bone formation (88)	◼ Uncoupling of osteoblast and osteoclast activity (56–58) ◼ Simultaneous occurrence of bone erosions and new bone formation (93–96)
THERAPEUTIC IMPACT ON RADIOGRAPHIC PROGRESSION	◼ TNF inhibitors reduce spinal radiographic progression (63) ◼ Similar reductions in radiographic progression were achieved with the anti-IL-17 agent secukinumab (104) and the dual IL-17A/F inhibitor bimekizumab (70) ◼ No efficacy of anti-IL-23 agents on spinal progression (128–130)	◼ Anti-IL23 (131–133) and anti-IL17 (116–118) agents have demonstrated benefit in reducing axial radiographic progression ◼ The anti–IL-12/23 agent ustekinumab has been shown to inhibit radiographic progression (115) ◼ TNF inhibitors have been shown to reduce radiographic progression (97–101)
DISTINCT STRUCTURAL FEATURES	◼ The sacroiliac joints are primarily involved, typically presenting as symmetrical sacroiliitis (44) ◼ Active osteitis of the vertebrae may follow sacroiliac joint involvement (45, 50) ◼ Active inflammatory lesions evolve into fat metaplasia (49), followed progressively by new bone formation in the form of fine, thin syndesmophytes (51) ◼ Progressive new bone formation ultimately results in ankylosis, leading to the characteristic “bamboo spine” appearance (51, 53)	◼ Peripheral erosive phenotype as the primary structural manifestation (107, 113) ◼ Exuberant new bone formation with a “fuzzy” appearance of juxta-articular bone (90) ◼ Metaphyseal periostitis (“ivory phalanx”) and joint subluxations (“pencil-in-cup” deformity) (91) ◼ Axial involvement in PsA may affect the spine (92) and SI joints (5) independently ◼ Bulky, irregular para-syndesmophytes, often involving the cervical spine (92)

TNF-α, (tumor necrosis factor-alpha); IL-17A, (interleukin−17A); IL-23, (interleukin−23); RANKL, (receptor activator of nuclear factor kappa B ligand); RANK, (receptor activator of nuclear factor kappa B); axSpA, (axial spondyloarthritis); PsA, (psoriatic arthritis).

## 6 Conclusions and final remarks

While significant advancements have been made in understanding the molecular pathways underlying bone remodeling in axSpA and PsA, the intricate interplay of inflammatory and mechanical factors remains incompletely elucidated. This review not only highlights the shared cytokine-driven processes of bone erosion and neoformation but also underscores the distinct pathogenetic and phenotypic differences between these two diseases. These distinctions call for a more nuanced differential definition, enhanced assessment tools, and tailored, disease- specific therapeutic strategies. One particularly compelling observation is the differential impact of cytokine networks, with a relative predominance of TNFα in axSpA and a greater reliance on IL-17, IL-22, and IL-23 pathways in PsA. This divergence is reflected in the clinical efficacy of targeted treatments, as IL-23 inhibitors have shown promising results in PsA but have failed to demonstrate efficacy in axSpA. These findings challenge the notion of a “one- size-fits-all” approach to managing spondyloarthtitis and emphasize the need for context- specific therapeutic paradigms. Moreover, the apparent dichotomy in bone remodeling, ankylosis and erosive lesions in axSpA versus the uncoupled yet concurrent processes of erosion and neoformation in PsA, warrants further exploration. A deeper understanding of how these processes is regulated and interconnected at the molecular and cellular levels could elucidate the mechanisms underlying disease progression and clinical heterogeneity. Future research priorities should include:

- Elucidating the mechanistic pathways that govern the transition between bone erosion and neoformation.
- Investigating the role of mechanical stress and biomechanical forces in modulating cytokine-driven remodeling processes.
- Assessing the long-term impact of both current and novel therapeutic agents on structural progression and functional outcomes.

Such efforts hold the potential to advance truly personalized medicine, equipping rheumatologists with the tools to refine therapeutic strategies that not only mitigate inflammation but also address the fundamental bone remodeling abnormalities driving disease burden, disability, and societal costs. Ultimately, addressing these critical gaps in knowledge will be essential to revolutionize the management of axSpA and PsA. By transitioning from reactive to proactive approaches, we can aim to preserve bone integrity, improve patient quality of life and reduce the significant socioeconomic impact of these chronic conditions.
